# Affinity of Serum Albumin and Fibrinogen to Cellulose, Its Hydrophobic Derivatives and Blends

**DOI:** 10.3389/fchem.2019.00581

**Published:** 2019-09-06

**Authors:** Rupert Kargl, Matej Bračič, Matic Resnik, Miran Mozetič, Wolfgang Bauer, Karin Stana Kleinschek, Tamilselvan Mohan

**Affiliations:** ^1^Laboratory for Characterization and Processing of Polymers, Faculty of Mechanical Engineering, University of Maribor, Maribor, Slovenia; ^2^Faculty of Technical Chemistry, Chemical and Process Engineering, Biotechnology, Institute of Paper, Pulp and Fibre Technology (IPZ), Graz University of Technology, Graz, Austria; ^3^Department of Surface Engineering and Optoelectronics, Jožef Stefan Institute, Ljubljana, Slovenia; ^4^Faculty of Technical Chemistry, Chemical and Process Engineering, Biotechnology, Institute of Inorganic Chemistry, Graz University of Technology, Graz, Austria

**Keywords:** cellulose acetate, ethyl cellulose, fibrinogen, albumin, hydrophilicity, quartz crystal microbalance, protein adsorption

## Abstract

This work describes the preparation of spin-coated thin polymer films composed of cellulose (CE), ethyl cellulose (EC), and cellulose acetate (CA) in the form of bi- or mono-component coatings on sensors of a quartz crystal microbalance with dissipation monitoring (QCM-D). Depending on the composition and derivative, hydrophilicity can be varied resulting in materials with different surface properties. The surfaces of mono- and bi-component films were also analyzed by atomic force microscopy (AFM) and large differences in the morphologies were found comprising nano- to micrometer sized pores. Extended protein adsorption studies were performed by a QCM-D with 0.1 and 10 mg mL^−1^ bovine serum albumin (BSA) and 0.1 and 1 mg mL^−1^ fibrinogen from bovine plasma in phosphate buffered saline. Analysis of the mass of bound proteins was conducted by applying the Voigt model and a comparison was made with the Sauerbrey wet mass of the proteins for all films. The amount of deposited proteins could be influenced by the composition of the films. It is proposed that the observed effects can be exploited in biomaterial science and that they can be used to extent the applicability of bio-based polymer thin films composed of commercial cellulose derivatives.

## Introduction

Synthetic and bio-based polymeric biomaterials are widely used for biomedical devices including those which are in contact with human blood, plasma, serum, or protein solutions. Applications for such materials are, among others, bio-separation techniques (Jungbauer, 2005) such as dialysis (Roumelioti et al., 2018), affinity chromatography (Burnouf and Radosevich, 2001), and electrophoresis (Rocco, 2005). For most of these applications knowledge on the interaction of blood, plasma, or serum proteins with the surfaces of materials is important. This knowledge allows drawing conclusion about the biocompatibility (Wang et al., 2013), coagulative properties (Vikinge et al., 2000), or separation performance (Zou et al., 2001) and finally determines their applicability. Commercial and research based separation columns or disposable biomedical materials are usually composed of very different synthetic or bio-based polymers and their derivatives. Among these bio-based polymers are cellulose and its esters and ethers (Klemm et al., 2005; Arca et al., 2018). Many authors therefore investigated the interaction of proteins with surfaces of polymers and elucidated its relation to hydrophilicity (Fujimori et al., 1998; Alves et al., 2010), charge (Edwards et al., 2012), morphology or solvation (Lu et al., 2007). Such studies were also conducted for cellulose and surface modified materials composed of it (Solin et al., 2019). Investigations on the interaction of proteins also included those with cellulose derivatives. Examples are published by Lv et al. (2017) who studied the protein binding and performance of cellulose nano-crystal modified cellulose acetate membranes and found that less albumin adsorbs with higher crystal content. Raghuwanshi et al. (2017) describe the interaction of immunoglobulins (IgG) at the solid liquid cellulose interface and Hong et al. (2008) used cellulose to efficiently separate proteins. Many studies describe the use of ethyl cellulose (EC) as particles, capsules or coatings for the controlled release and targeted delivery of drugs (Graves et al., 2005; Rogers and Wallick, 2012; Adebisi and Conway, 2014; Fan et al., 2014). Polymer blends with EC were used to control and retard the release of drugs from polymer based nanoparticles (Lecomte et al., 2005; Hasan et al., 2007). Although it is essential for drug delivery and the application of EC as biomaterial, adsorption of proteins on EC was surprisingly less frequently studied (Casilla and Eley, 1975; Bruck, 1976; Abu-Lzza and Lu, 1998; Hoffart et al., 2007). This work therefore aims at investigating and comparing the interaction of the proteins bovine serum albumin (BSA) and fibrinogen (FIB) on commercially available celluloses and their derivatives in the form mono- and bi-component thin films using a quartz crystal microbalance with dissipation monitoring (QCM-D). Both proteins are essential in the assessment of biocompatibility and blood coagulation and any material composed of these polymers that comes into contact with full blood or plasma will be initially covered by these proteins as confirmed by QCM-D and surface plasmon resonance (SPR) (Mohan et al., 2013, 2014, 2017). Films are prepared by spin coating trimethylsilyl cellulose (TMSC) in combination with cellulose acetate (CA) or ethyl cellulose (EC) on sensors of a QCM-D. Cleavage of the trimethylsilyl protecting groups by acid vapor hydrolysis results in blends composed of cellulose and either contain CA or EC. Bi-component blends were also prepared from CA-EC. Static water contact angle measurements are performed to assess the hydrophilicity of the blend films and morphological studies by atomic force microscopy (AFM) are included. Understanding protein adsorption on mono- and bi-component films of cellulose and its derivatives should allow for a basic understanding of their surface properties and result in alternative applications for these bio-based polymers.

## Materials and Methods

### Film Preparation

Trimethylsilyl cellulose (TMSC, DS_TMS_: 2.8, Mw: 149 kDa, derived from Avicel PH-101) was purchased from Thüringisches Institut für Textil- und Kunststoffforschung e.V. (TITK, Rudolstadt, Germany). Cellulose acetate (CA, acetyl content 38 wt.% Mw: 30 kDa, Sigma-Aldrich, Austria) and ethyl cellulose (EC, viscosity 100 cP, 5% in toluene/ethanol 80:20, extent of labeling: 48% ethoxyl, Sigma-Aldrich Austria) were used as received. For mono-component films, polymers were dissolved at 1 wt.% in tetrahydrofuran p. a. (THF, Sigma Aldrich Austria). For bi-component films of CA-EC both polymers were dissolved each at 0.5 wt.% in the same solvent and solution. For blend films containing cellulose (CE) the TMSC precursor concentration in the spinning solution was 1.13 wt.% since cleavage and removal of TMS groups during regeneration into cellulose causes a mass loss of 55.8 wt.%. Concentration of the other component (CA or EC) was 0.5 wt.%. Films were made by dropping 50 μL polymer solution on the static QCM-D crystals with a gold electrode layer (QSX-301, LOT-Oriel, Germany) and spin coating them at 4,000 rpm and an acceleration of 2,500 rpm sec^−1^. Prior to coating QCM-D crystals were immersed into a mixture of H_2_O/H_2_O_2_ (30 wt.%)/NH_4_OH (5:1:1; v/v/v) for 10 min at 70°C, then immersed in a “piranha” solution containing H_2_O_2_ (30 wt.%)/H_2_SO_4_ (1:3; v/v) for 40 s, and then rinsed with water and finally blown dry with N_2_ gas.

Films obtained were composed of TMSC, CA, EC, TMSC-CA 1.1:0.5 w/w, TMSC-EC 1.1:0.5 w/w, and CA-EC 1:1 w/w. Films containing TMSC were placed into a 20 mL polystyrene petri-dish (4 cm in diameter) and 2 mL 10 wt.% HCl was dropped next to the QCM-D crystals and the petri-dish was covered with its cap. HCl vapors deprotected the cellulose hydroxyl groups by acid catalyzed hydrolysis of TMS groups leading to a mass loss of 55.6 wt.% for TMSC within 30 min and cellulose (CE) was obtained in the film (Mohan et al., 2017) resulting in a polymer blend mass ratio of 1:1 (CE-EC; CE-CA). Bi-component films then containing cellulose instead of TMSC were labeled CE-CA, CE-EC.

### Atomic Force Microscopy

Surface morphology was analyzed by Atomic Force Microscopy (AFM, Solver PRO, NT-MDT, Moscow, Russia) in semi-contact mode in air. The sample surfaces were scanned by a standard silicon cantilever with a force constant of 16 N/m at a resonance frequency of 325 kHz. Cantilevers' tip radius was 10 nm, the tip length was 95 μm and the scan rate was set at 1.56 Hz. At least three different areas of each sample were measured. The average root mean square surface roughness (Sq) was calculated from representative images on 5 × 5 μm^2^ areas, with the Nova AFM software provided be the manufacturer.

### Infrared Spectroscopy

Infrared transmission spectra (Perkin Elmer FTIR Spectrum GX spectrometer with ATR-IR attachment and diamond crystal) were measured on the coated QCM-D crystals at room temperature and humidity with 16 scans and a resolution of 4 cm^−1^.

### Contact Angle

Static water contact angle measurements were performed using an OCA15+ contact angle measurement system from Dataphysics (Germany). All measurements were conducted at room temperature and humidity without conditioning of the environment on at least two independent surfaces on QCM-D crystals with a drop volume of 3 μl. Each contact angle value was the average of at least four drops of liquid per surface. For contact angle measurements water (>18 MΩ cm) from a Milli-Q-water system (Millipore, USA) was used.

### Quartz Crystal Microbalance

A QCM-D instrument (model E4) from Q-Sense (Gothenburg, Sweden) was used. The instrument simultaneously measures decreases/increases in the resonance frequency (Δ*f* ) and increases/decreases in energy dissipation (Δ*D*) when the mass of an oscillating piezoelectric crystal increases/decreases due to deposition/removal of material. Dissipation refers to the frictional losses that lead to damping of the oscillation depending on the viscoelastic properties of the material. For a rigid adsorbed layer that is fully coupled to the oscillation of the crystal, Δ*fn* is given by the Sauerbrey Equation (1) (Sauerbrey, 1959).

(1) $$\begin{array}{r} \Delta m=C\frac{\Delta f_{n}}{n} \end{array}$$

where Δ*f*_*n*_ is the observed frequency shift, C is the Sauerbrey constant (−0.177 mg Hz^−1^ m^−2^ for a 5 MHz crystal), *n* is the overtone number (*n* = 1, 3, 5, etc.) and Δ*m* is the change in mass of the crystal due to the adsorbed or desorbed layer. The mass of a soft (i.e., viscoelastic) film is not fully coupled to the oscillation and the Sauerbrey relation is not valid since energy is dissipated in the film during the oscillation. The energy dissipation (*D*) is defined as (2):

(2) $$\begin{array}{r} D=\frac{E_{diss}}{2\pi E_{stor}} \end{array}$$

where *E*_*diss*_ is the energy dissipated and *E*_*stor*_ is the total energy stored in the oscillator during one oscillation cycle.

For protein adsorption studies, bovine serum albumin (BSA, 0.1 and 10 mg mL^−1^) and fibrinogen from bovine plasma (FIB, 0.1 and 1 mg mL^−1^) were gently dissolved in a 10 mM PBS buffer at pH 7.4 and room temperature. Both proteins and the PBS were from Sigma Aldrich, Austria.

Quartz crystal microbalance with dissipation monitoring (QCM-D) crystals coated with the thin films were mounted in the QCM-D flow cell and equilibrated with MilliQ-water and 10 mM PBS buffer solution until a stable frequency signal was established. BSA or FIB were pumped through the QCM-D cell for 40 min, followed by the PBS solution for 25 min and water for 30 min. The flow rate was kept at 0.1 mL min^−1^ and the temperature at 21°C throughout all experiments. All adsorption experiments were performed in two parallels and a mean value of the third overtone of dissipation (*D*_3_) and frequency (*f*_3_) was displayed. The overtones (*f*_3_-*f*_11_) were separately displayed for one measurement per sample. ***Viscoelastic modeling:*** The viscoelastic Voigt model was applied for calculating the adsorbed mass (Γ_QCM_), film thickness (*h*_f_), viscosity (η_f_), and elastic shear modulus (μ_f_) of the adsorbed BSA and FIB layer. In this model, the adsorbed layer was treated as a viscoelastic layer between the quartz crystal and a semi-infinite Newtonian liquid layer. More details on the Voigt modeling can be found elsewhere (Voinova et al., 1999; Höök et al., 2001). For data evaluation or fitting the different overtones (*n* = 3, 5, 7, 9, 11, and 13) of frequency and dissipation were used. All calculations were carried out using the software package QTools 3.0.12 (Q-Sense). The fitting parameters used for the modeling are: viscosity, from 1 × 10^−4^ to 0.01 N·s·m^−2^; elastic shear modulus, from 1 × 10^4^ to 1 × 10^8^ N·m^−2^; and thickness, from 1 × 10^−10^ to 1 × 10^−6^ m. It is worth noting that the values of *h*_f_ and ρ_f_ were not independent variables. In order to calculate the effective thickness and adsorbed mass (Equation 3), the density ρ_f_ values was varied between 1,000 and 1,130 kg m^−3^. It turned out that no mass change for BSA/FIB coated layer occurred by changing the density value and therefore a density (ρ_f_) of 1,000 kg m^−3^ was used for all calculations (Equation 3).

(3) $$\begin{array}{r} \Gamma_{\text{QCM}}=h_{\text{f}}\rho_{\text{f}} \end{array}$$

## Results and Discussion

### Morphology and Composition of the Films

**Figure 1:**
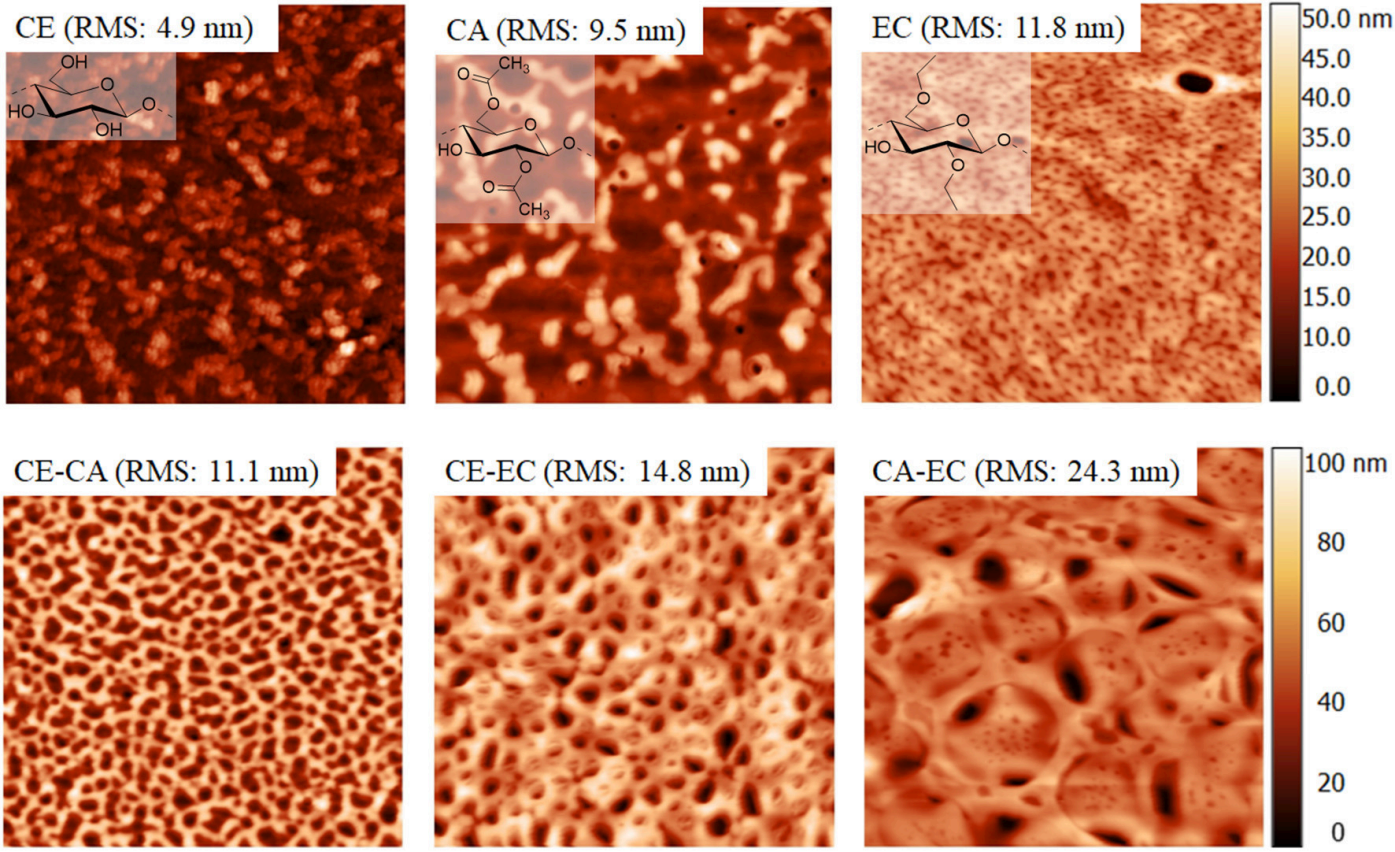
Atomic force microscopy height image of cellulose (CE), cellulose acetate (CA), ethyl cellulose (EC), and their blends. Image size is 5 × 5 μm^2^.

Figure 1 shows 5 × 5 μm^2^ AFM height images of all films investigated. According to root mean square roughness calculations the smoothest coatings are obtained from cellulose. Significant differences in structural features can be seen. Most notably a pore sizes in the nanometer range and a concomitant surface roughness increase for blend films and a maximum for CA-EC is observed. Interestingly, pore size distribution is relatively uniform for the films. Care must be taken that the surface morphology and accessibility of the films for water and protein are considered with respect to the QCM-D and contact angle results. Since these materials are very different form a morphological and chemical point of view, attempts to perfectly quantify the amount of retained protein by QCM-D can be challenging (Vikinge et al., 2000; Reviakine et al., 2011).

**Figure 2:**
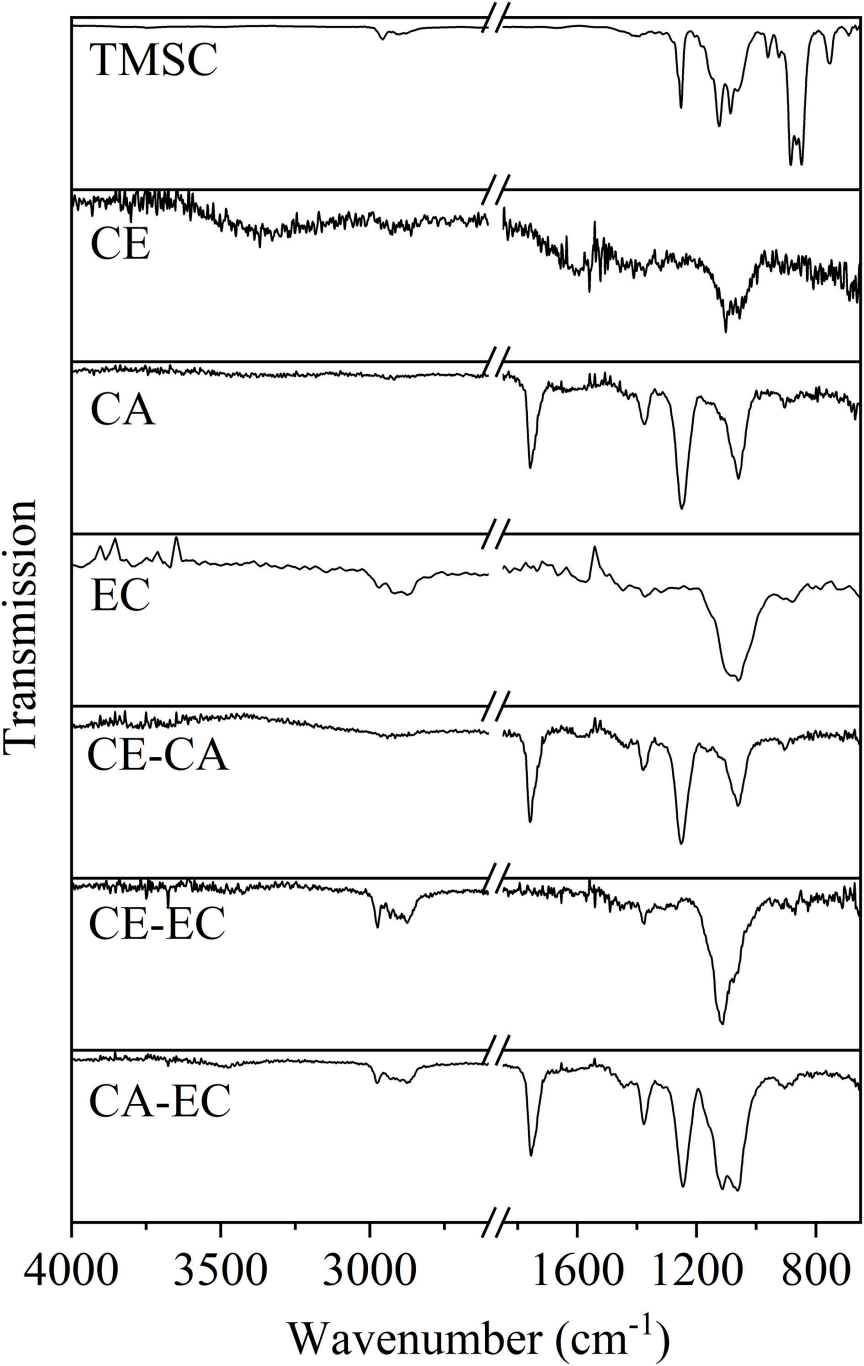
Infrared transmission spectra for trimethylsilyl cellulose (TMSC), cellulose (CE), cellulose acetate (CA), ethyl cellulose (EC), and blends thereof on QCM-D gold crystals.

The infrared transmission spectra of the films are given in Figure 2. For clarity reasons also a neat trimethylsilyl cellulose film (TMSC) is shown. The materials show all expected peaks according to literature values (Shi et al., 2008; Kargl et al., 2012). Most importantly for films containing cellulose (CE) a deprotection of the trimethyl silyl groups could be confirmed by the absence of peaks at 1,252 and 848 cm^−1^ representative for the Si-C vibrations (Mohan et al., 2011).

### Hydrophilicity of the Films

**Figure 3:**
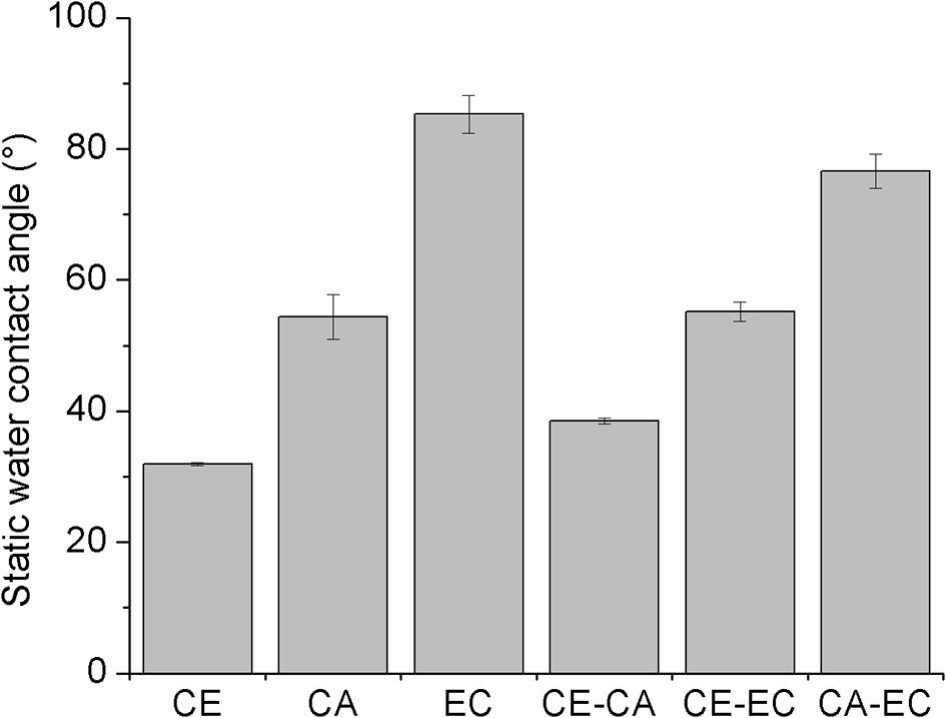
Static water contact angles of spin-coated films composed of cellulose (CE), cellulose acetate (CA), and ethyl cellulose (EC) or their blends (CE-CA, CE-EC, CA-EC). All blend films are composed of 50 wt.% for each polymer.

Figure 3 depicts the static water contact angles of mono- and bi-component thin films. As expected from the molecular structure, cellulose (CE) is the most hydrophilic polymer with a contact angle of 31.9 ± 0.2°. Compared to ethyl cellulose (EC, 85.3 ± 2.9°), cellulose acetate (CA, 54.4 ± 3.4°) is a less hydrophobic material owing to its ability to act as a hydrogen bond acceptor over the electron lone pairs of the carboxyl oxygen atoms and as a donor and acceptor at unsubstituted hydroxyl groups. Blending hydrophobic cellulose derivatives in bi-component films with cellulose, results in more hydrophilic surfaces (CE-CA 38.5 ± 0.4°) confirming the exposure of cellulose R-OH groups and the ability to interact with larger amounts of water. Blending EC with CA however, gives surfaces with an intermediate hydrophobicity (CA-EC, 76.6 ± 2.6°). A static water contact angle of 55.2 ± 1.5° is observed by combining the most hydrophilic material CE with the most hydrophobic cellulose derivative (EC). As a result, a variety of thin films composed of the glycan chain of cellulose but with different substituents is available, whose hydrophilicity and number of available hydrogen bond acceptor and donor groups can be adjusted by simple blending of the polymers. These films were subjected to interaction studies with different concentrations of bovine serum albumin and fibrinogen from bovine plasma in phosphate buffered saline at pH 7.4 and 21°C.

### Interaction of Bovine Serum Albumin With the Films

**Figure 4:**
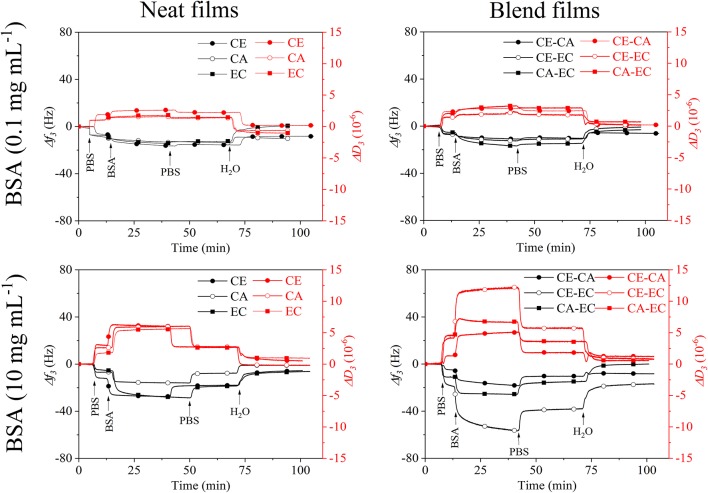
QCM-D frequency (*f*_3_) and dissipation change (*D*_3_) during rinsing with 0.1 and 10 mg mL^−1^ bovine serum albumin over cellulose (CE), cellulose acetate (CA), and ethyl cellulose (EC) and their 50:50 wt.% blends (CE-CA, CE-EC, CA-EC) in PBS followed by rinsing with PBS and water.

Figure 4 depicts frequency (*f*_3_) and dissipation (*D*_3_) changes from QCM-D experiments on CE, CA, and EC films and the blends CE-CA, CE-EC, and CA-EC. A BSA concentration of 0.1 mg mL^−1^ (upper row) and 10 mg mL^−1^ (lower row) in PBS (pH 7.4) was used. Initial rinsing of the films with buffer reduces the frequency and increases the damping of the oscillation due to a higher density of PBS compared to water. Subsequent introduction of the protein solution results in a negative frequency and positive dissipation shift which is caused by: (a) adsorption of the protein and (b) a higher viscosity and density of the solution in contact with the film on the crystal. For 0.1 mg mL^−1^ BSA no significant difference in the *f*_3_ and *D*_3_ response can be observed for neat and blend films during contact with the protein and rinsing with PBS or water. Final values after rinsing almost reach the initial stage. For 10 mg mL^−1^ BSA significant differences are observed for neat films with CA showing a lower response than CE and EC for all stages of adsorption and rinsing. QCM-D signals on blend films a significantly different for CE-EC, the material which contains a combination of the most and the least hydrophilic polymer. This feature obviously allows for a better interaction of the protein with the film.

A spread in the response of the different overtones of oscillation and dissipation (Figures S1, S2 is observed for all films at 10 mg mL^−1^ BSA demonstrating the viscoelastic behavior of the protein layer and solution. This spread almost disappears upon the final rinsing with water leading to the qualitative statement that the retained protein layer has a more compact, less swollen form on all films.

**Figure 5:**
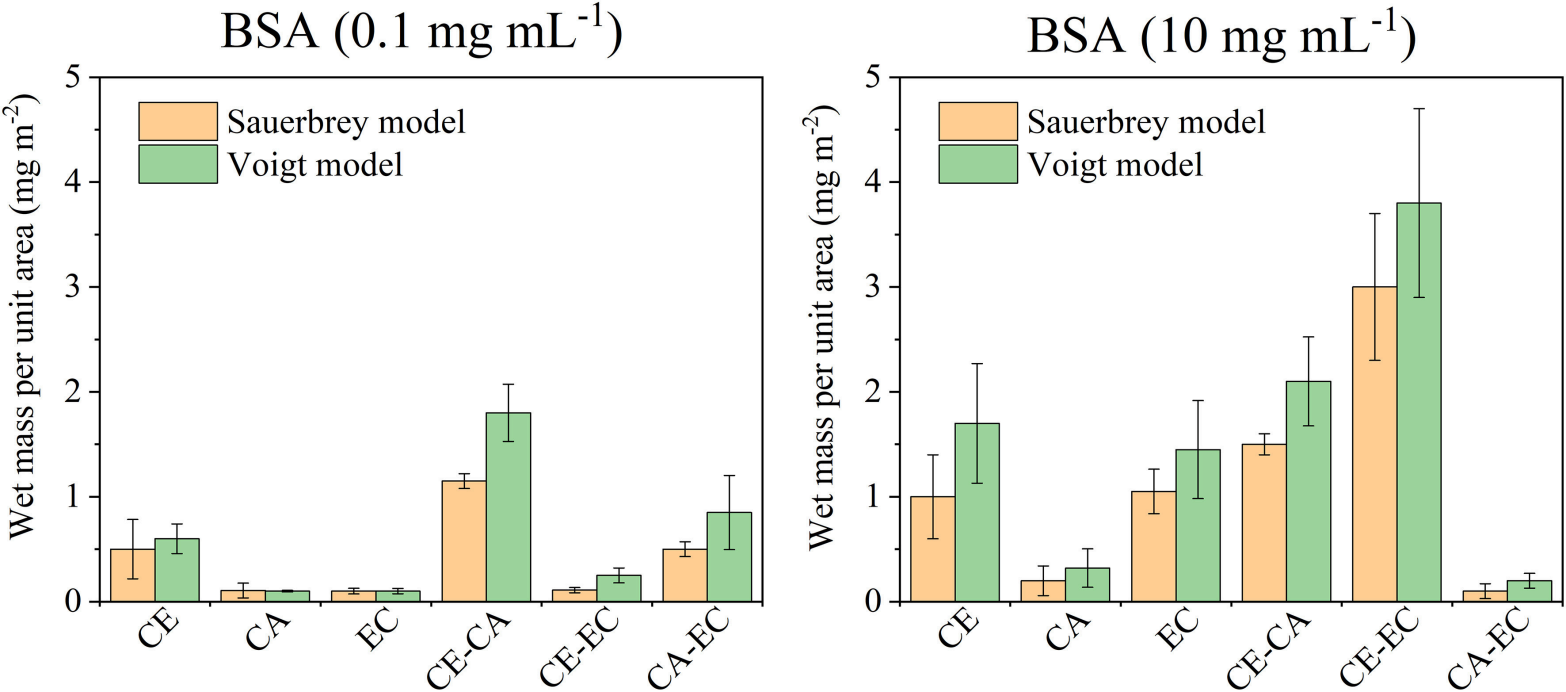
Wet mass of retained BSA per unit area as calculated from the Sauerbrey equation or Voigt model after films were in contact with 0.1 or 10 mg mL^−1^ protein in PBS and subsequently rinsed with PBS and water. Deposited protein masses are shown for cellulose (CE), cellulose acetate (CA), ethyl cellulose (EC), and their blends (CE-CA; CE-EC, CA-EC).

A summary of the frequency and dissipation shifts after BSA adsorption and final rinsing with water is given in Figure 5, displaying the Sauerbrey and Voigt wet mass of adsorbed protein per unit area. For a low protein concentration of 0.1 mg mL^−1^ only CE and blends composed of CA-EC and CE-CA have a significantly higher protein affinity. For 10 mg mL^−1^ BSA the highest affinity was found for blends composed of cellulose and its ethyl derivative. Again, it is hypothesized that this mixed blend of the most hydrophilic with the most hydrophobic material allows BSA to attached more efficiently and irreversibly. The Voigt wet mass per unit area, as calculated by modeling the data from six overtones, is higher for all measurements but follows the same trend as the Sauerbrey mass calculated from the linear relation of frequency and deposited mass. It is important to note that the same surface area is assumed for all films for this calculation.

### Interaction of Fibrinogen With the Films

**Figure 6:**
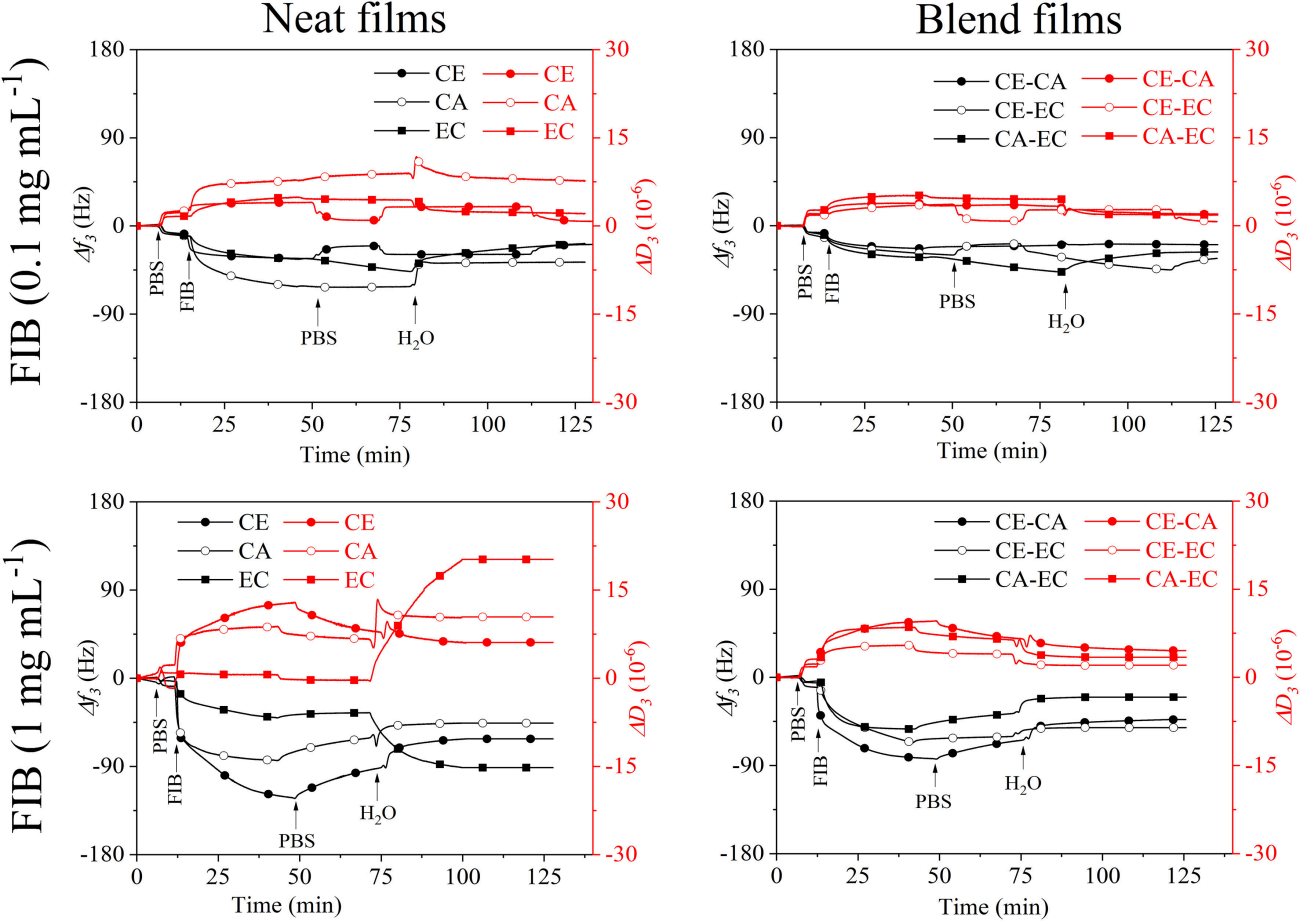
QCM-D frequency (*f*_3_) and dissipation change (*D*_3_) during rinsing with 0.1 and 1 mg mL^−1^ fibrinogen (FIB) from bovine plasma in PBS over cellulose (CE), cellulose acetate (CA), and ethyl cellulose (EC) and their 50:50 wt.% blends (CE-CA, CE-EC, CA-EC) followed by rinsing with PBS and water.

Fibrinogen adsorption on biomaterials is essential in processes involving blood clot formation. The binding of this protein at concentrations similar to those found in human plasma is therefore of interest in biomaterial research. Figure 6 shows the interaction of fibrinogen in PBS (0.1 and 1 mg mL^−1^) with neat (CE, CA, EC) and blend (CE-CA, CE-EC, CA-EC) polysaccharide thin films.

At 0.1 mg mL^−1^ already differences in the affinity of FIB toward the cellulose derivatives can be observed. The largest frequency shift and presumably strongest interaction is seen on cellulose acetate (CA). Upon rinsing with PBS and water, all films show a similar response in *f*_3_. CA however gives higher *D*_3_ leading to the conclusion than only minor amounts of FIB are retained on all films with a maximum on cellulose acetate.

When 1 mg mL^−1^ fibrinogen in PBS are introduced the strongest QCM-D response can be found for cellulose films. Upon rinsing with PBS minor desorption is observed. Rinsing with water however reveals that only on the most hydrophobic ethyl cellulose (EC) larger amounts of FIB are retained that incorporate water and substantially increase the *D*_3_ value. Swelling and water retention is so strong that *f*_3_ even decreases in contrast to all other films. Since pure water is used for rinsing, this also strongly increases the osmotic pressure in the film and adsorbed layer upon ion exchange, resulting in such a pronounced swelling. Interestingly in blends with other materials this behavior is not observed for EC demonstrating that the second polymer cellulose or its acetate ester in the bi-component film suppresses FIB interaction and retention. The spread in the response of the different overtones of oscillation and dissipation (Figures S3, S4) is more pronounced for 1 mg mL^−1^ FIB but is significantly lowered after rinsing with water, similar to the results from BSA.

**Figure 7:**
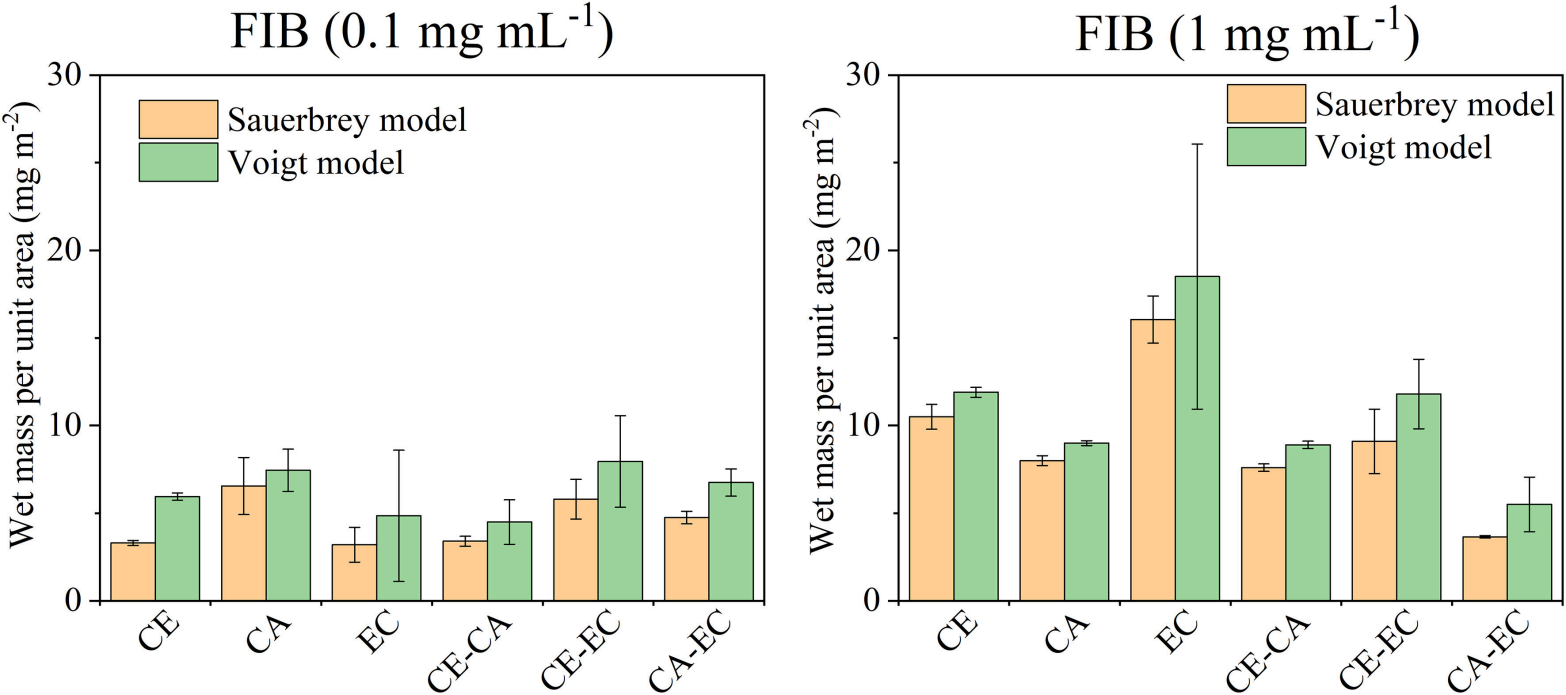
Wet mass of retained fibrinogen (FIB) per unit area as calculated from the Sauerbrey equation or Voigt model after films were in contact with 0.1 or 1 mg mL^−1^ protein in PBS and subsequently rinsed with PBS and water. Deposited protein masses are shown for cellulose (CE), cellulose acetate (CA), ethyl cellulose (EC), and their blends (CE-CA, CE-EC, CA-EC).

Figure 7 depicts a summary of the retained fibrinogen wet masses per unit area for all films investigated. The Sauerbrey relation and the Voigt model were used to calculate these wet masses. For 0.1 mg mL^−1^ fibrinogen the largest retention can be found on cellulose acetate even though overall the amounts are similarly low for all films. Fibrinogen deposition is however more significant than serum albumin retention on the same materials in terms of overall mass which can also be attributed to the much higher molecular mass of FIB. As already discussed above, significant differences are observed for 1 mg mL^−1^ fibrinogen on ethyl cellulose (EC) which is also the most hydrophobic materials. Again, this significant impact of EC diminished in blends with the other polymers.

## Conclusion

It was shown that the commercial cellulose derivatives trimethylsilyl cellulose, cellulose acetate and ethyl cellulose can be blended and shaped into thin films by spin coating on sensors of a quartz crystal microbalance. The trimethylsilyl cellulose in the films can be regenerated to cellulose by exposure to vapors of hydrochloric acid as confirmed by infrared transmission measurements. The surface morphology and roughness of blend films containing a mass ratio of 50% of each polymer strongly varies between different blends. Nano- to micrometric features with relatively regular pore sizes can be obtained. Those films that contain cellulose are hydrophilic and hydrophobicity increases for cellulose acetate and is the highest for ethyl cellulose. The wetting of blend films with water reflects the composition and the static water contact angles are on average in between those of the single component materials. Quartz crystal microbalance with dissipation monitoring showed, that blend films composed of cellulose and ethyl cellulose significantly bind more bovine serum albumin than all other films at an initial protein concentration of 10 mg mL^−1^. Fibrinogen is however retained most significantly on the most hydrophobic polymer ethyl cellulose, already at initial concentrations of 1 mg mL^−1^. Sauerbrey and Voigt models of the retained protein masses reveal similar amounts which is also reflected in a minor spread of the overtones of oscillation.

## Data Availability

The datasets generated for this study are available on request to the corresponding author.

## Author Contributions

RK contributed to experimental planning, data analysis and writing. MB performed the QCM-D experiments, analyzed and interpreted the data and contributed to the manuscript. MR performed the atomic force microscopy measurements and calculation. MM interpreted the surface analytical results and contributed to the writing. WB contributed to the overall concept and manuscript revision. KS guided the work, scientifically interpreted it and contributed to manuscript writing. TM oversaw all experimental planning, data analysis, and writing.

### Conflict of Interest Statement

The authors declare that the research was conducted in the absence of any commercial or financial relationships that could be construed as a potential conflict of interest.
