# Supplementary material for: Affinity of Serum Albumin and Fibrinogen to Cellulose, Its Hydrophobic Derivatives and Blends
Source: Front Chem. 2019 Sep 6;7:581. doi: 10.3389/fchem.2019.00581 (PMC6743410; doi:10.3389/fchem.2019.00581)
Supplement: Supplementary file 1 [file Data_Sheet_1.docx]

**Supplementary material**

Affinity of serum albumin and fibrinogen to cellulose, its hydrophobic derivatives and blends

Rupert Kargl^1,2,*^, Matej Bračič^1^, Matic Resnik^3^, Miran Mozetič^3^, Wolfgang Bauer^2^, Karin Stana Kleinschek^1,4^, Tamilselvan Mohan^1^

^1^Laboratory for Characterization and Processing of Polymers, Faculty of Mechanical Engineering, University of Maribor, Smetanova ulica 17, Maribor, Slovenia.

^2^Institute of Paper, Pulp and Fibre Technology (IPZ), Graz University of Technology, Inffeldgasse 23, A-8010 Graz, Austria.

^3^Department of Surface Engineering and Optoelectronics, Jožef Stefan Institute, Jamova cesta 39, SI-1000 Ljubljana, Slovenia.

^4^Institute of Inorganic Chemistry, Graz University of Technology, Stremayrgasse 9/V, A-8010 Graz, Austria.

*** Correspondence:**Corresponding Author
rupert.kargl@um.si

Keywords: cellulose acetate, ethyl cellulose, fibrinogen, albumin, hydrophilicity, quartz crystal microbalance, protein adsorption





**Figure S1:** QCM-D frequency and dissipation change (*f_3_, f_5_, f_7_, f_9_, f_11_*) during rinsing with 0.1 and 10 mg ml^-1^ bovine serum albumin (BSA) over cellulose (CE), cellulose acetate (CA), and ethyl cellulose (EC) in PBS followed by rinsing with PBS and water.





**Figure S2:** QCM-D frequency and dissipation change (*f_3_, f_5_, f_7_, f_9_, f_11_*) during rinsing with 0.1 and 10 mg ml^-1^ bovine serum albumin (BSA) over cellulose (CE), cellulose acetate (CA), and ethyl cellulose (EC) 50:50 wt.% blends (CE-CA; CE-EC; CA-EC) in PBS followed by rinsing with PBS and water.

**

**

**Figure S3:** QCM-D frequency and dissipation change (*f_3_, f_5_, f_7_, f_9_, f_11_*) during rinsing with 0.1 and 1 mg ml^-1^ fibrinogen (FIB) from bovine plasma over cellulose (CE), cellulose acetate (CA), and ethyl cellulose (EC) in PBS followed by rinsing with PBS and water.

**

**

**Figure S4:** QCM-D frequency and dissipation change (*f_3_, f_5_, f_7_, f_9_, f_11_*) during rinsing with 0.1 and 1 mg ml^-1^ fibrinogen (FIB) from bovine plasma over cellulose (CE), cellulose acetate (CA), and ethyl cellulose (EC) 50:50 wt.% blends (CE-CA; CE-EC; CA-EC) in PBS followed by rinsing with PBS and water.
